# Spray-Dried Powders of Casein-Encapsulated Rutin Stabilized With Sugars for the Enhancement of Intestinal Drug Solubility

**DOI:** 10.1155/adpp/9952737

**Published:** 2025-03-02

**Authors:** Helmy Yusuf, Sinta Choirunissa Fitriana, Ni Luh Eradeasty Putri Darmawan, Revalida Ainun Nisa, Retno Sari, Dwi Setyawan

**Affiliations:** ^1^Department of Pharmaceutical Sciences, Faculty of Pharmacy, Airlangga University, Surabaya 60115, Indonesia; ^2^Pharmaceutics and Delivery Systems for Drugs, Cosmetics, and Nanomedicine Research Group, Department of Pharmaceutical Sciences, Faculty of Pharmacy, Airlangga University, Surabaya 60115, Indonesia

## Abstract

Numerous therapeutic potentials of rutin (RUT) including cardioprotective, neuroprotective, and antihypertension activities have attracted many studies to bring it into clinical use. RUT is phytochemically derived from plants such as apples and tea. It is poorly soluble and very sensible to acidic pH in the stomach environment which leads to conceded oral bioavailability. In contrast, RUT is better soluble in basic environment, thus, encapsulating RUT within enteric microparticles (RUT-MP) using casein (CAS) resolved such problems. The encapsulation by spray-drying employed sugars (lactose, sucrose, and maltodextrin) as bulking agents and for stabilization of the amorphous drug. The developed RUT-MP formulations were prepared in two groups i.e., lower and higher RUT concentrations. The solid states were studied by X-ray diffraction (XRD), differential thermal analysis (DTA) and scanning electron microscopy (SEM). Solubility tests were also carried out on the samples to examine the outcome of the engineered physical modification. The results showed that the RUT-MPs were spherical in morphology. The RUT was transformed into amorphous structure as suggested by the XRD and DTA results indicating that RUT was molecularly dispersed in the RUT-MP. There were no phase separations that occurred as confirmed by the DTA data. Solubility tests carried out on the RUT-MPs showed that the encapsulation with CAS in group with higher concentration of RUT prevented the drug against recovery of the crystallinity and phase separations. The solubility test revealed various substantial enhancements of RUT solubility of the RUT-MPs at pH 7.0. The highest enhancement of RUT solubility was 191,5-fold, with respect to pure RUT. The presence of sugars was beneficial as they improved the yield percentage and might have contributed to the prevention of nano-crystal aggregation which made them a determining aspect for the successful application of spray-dried encapsulation.

## 1. Introduction

To date, 70 different plant species have produced the flavonol glycoside RUT (3′,4′,5,7-tetrahydroxy flavone-3-rutinoside) [1, 2]. RUT has been extensively used in pharmaceutical applications and the development of dietary supplements and nutraceuticals because of its demonstrated pharmacological properties, which include cardioprotective, neuroprotective, and antihypertension effects [3–5].

Nevertheless, pharmaceutical formulators often face challenges in the oral administration since RUT is very hydrophobic molecule which make it poorly soluble in water at 0.0639 mg/L [6, 7]. Furthermore, RUT does not dissolve well in acidic environments [8]. When used orally, RUT solubility is restricted in low-pH environments such as the stomach, hence limiting its absorption and bioavailability [8, 9]. In alkaline settings with a higher pH, like the intestines, RUT is more soluble [7, 10]. This improved solubility in alkaline environments promotes improved absorption and dissolution in this segment of the digestive system [11, 12].

To increase bioavailability of RUT, different formulation techniques such as the use of solubilizers, solid dispersion or encapsulation are frequently investigated [13–15]. One particular interest is the enhancement of drug solubility through encapsulation. There are several materials that can be used for this encapsulation, including polymers, liposomes, and nanoparticles [16–18]. Considering that RUT is less soluble in acidic stomach environment and more soluble in basic intestine, the encapsulation strategy may be carried out by encapsulating drug with enteric carriers, in which their solubility being reliant on pH [19]. Carriers which are not soluble in acidic but soluble in alkaline environment can protect RUT particles in the stomach and increase the RUT solubility and absorption in the intestine. At the pH of the small intestine, the enteric carrier MP would disintegrate and release the drug at the absorption site [20].

A previous work described pH-responsive MP systems that offer a special platform for preparing homogeneous, spherical MP that can contain drugs with a variety of physicochemical characteristics [21, 22]. In contrast to a pure drug material, the poorly soluble drug in the amorphous form that the MP provided dissolved quickly in the basic pH [23].

Casein (CAS) has been considered as one of the most promising proteins to use as encapsulating carriers [24, 25]. CAS exhibit good safety, biocompatibility, and numerous binding sites [26, 27]. In the presented study, the enhancements of RUT solubility through encapsulation in pH-driven CAS MP were investigated. In addition, to avoid nano-crystal aggregation, a variety of bulking agents have been used during spray-drying [28]. Therefore, the use of sugars i.e., lactose (LAC), sucrose (SUC), and maltodextrin (MAL) as bulking agents were also studied.

The presented study suggests a new strategy to address this issue using pH-responsive CAS MP that, by delivering RUT to intestinal site, increase the bioavailability of RUT. In this manuscript, the formulations of RUT-CAS in form of MP (RUT-MP) were developed to enhance RUT solubility. The physical properties were investigated in terms of their morphology, thermal properties, drug crystallinity, and solubility of the encapsulated RUT.

RUT-MP offer multiple advantages over traditional enteric-coated formulations, including enhanced intestinal solubility, faster dissolution, targeted delivery, and potentially reduced gastrointestinal irritation. Nevertheless, to advance RUT-MP formulations toward clinical application, several critical studies are necessary. They include pharmacokinetics and pharmacodynamics assessments to determine bioavailability, metabolism, and excretion; in vitro cytotoxicity for safety evaluation and stability studies to ensure that the MP maintain their efficacy and safety over time under various storage conditions.

## 2. Materials and Methods

### 2.1. Materials

RUT (≥ 94%), CAS (from bovine milk), LAC, SUC, MAL, ethanol, and all other reagents were purchased from Sigma-Aldrich (Singapore).

### 2.2. Preparation of RUT-MP Formulations

The RUT-MP were prepared as follows: briefly several amounts of RUT, CAS, and sugars were weighed according to Table 1. RUT was dissolved in 50 mL of ethanol 96% and drop-wised to CAS solution in 50 mL of NaOH 1 N under constant stirring at 1000 rpm. In terms of using sugars as bulking agents, LAC/SUC/MAL was dissolved in 30 mL of aquadest. The sugar solution was then added to the mixture of RUT and CAS solutions. The resulting mixture was then diluted with aquadest to make a total volume of 250 mL solution and dried by spray-drying. Buchi B-290 Mini-Spray Dryer (Flawil, Switzerland) equipped with a high-performance cyclone was employed to produce the MP powders. Conditions of the spray-drying were as follows: the inlet temperature was 150°C, the outlet temperature was 90°C, the spraying pressure ranged from 5.0 to 5.8 mbar, the feed flow rate was 5 mL/min, the airflow rate was 320 L/h, and 90% of the air was used for aspiration. Prior to additional analysis, the acquired RUT-MP was collected in a tight container and kept at 25°C in a desiccator [29].

### 2.3. Drying Yield Determination

The entire dry powder of RUT-MP collected after the spray-drying procedure was divided by the initial quantity of components in the formula to get the percentage of drying yield. The following formula was used to get the yield percentage:

(1) $$\begin{array}{c} \%\text{Yield}=\frac{\text{total weight of dry powder}}{\text{total weight of initial formula}}\times 100\%. \end{array}$$

### 2.4. Scanning Electron Microscopy (SEM)

The RUT-MP dry powders morphology was characterized using scanning electron microscope (Phenom, USA). Dry powders were put and glued using adhesive to plates, and then fastened to specimen mounts. The samples were then subjected to sputter coating with a 5 nm thick layer of gold and analyzed [29].

### 2.5. X-Ray Diffractometry (XRD)

To characterize the crystallinity of the raw materials and RUT-MPs, a Philips scanning X-ray diffractometer (Philips X'Pert PRO; Panalytical, The Netherlands) was used. Samples were placed in a disc sample holder and were gently compressed and smoothed. Samples were analyzed at 25 mA and 40 kV, scanning rate of 2°/min step from 5° to 40° [30].

### 2.6. Differential Thermal Analysis (DTA)

A DTA instrument (Mettler Toledo FP 85, Switzerland) was utilized to identify the dry product of RUT-MP. Samples were put inside aluminum crucibles, and a heating rate of 10°C/min was used to conduct the DTA analysis throughout a temperature range of 30°C to 250°C [17].

### 2.7. Solubility Study

RUT-MP (500 mg or equivalent amount of RUT beyond its saturated concentration) were weighed and then placed in a vial containing 5 mL of CO_2_-free aquadest pH 7.0 ± 0.05. The total duration of the experiment was 6h, the speed of the stirring was 200 rpm, and the temperature of the medium was 37°C ± 0.5°C. Each solubility test was carried out in triplicate. The absorbances of raw RUT and RUT-MP formulations were assayed at 355 nm using a UV spectrophotometer.

### 2.8. Statistical Analysis

The statistical data analysis was carried out using the program GraphPad InStat (San Diego, CA). A parametric one-way ANOVA test was used to analyze the experimental data on solubility. 95% confidence limits were utilized to establish statistical significance.

## 3. Results and Discussions

### 3.1. Yield Percentage

**Figure 1:**
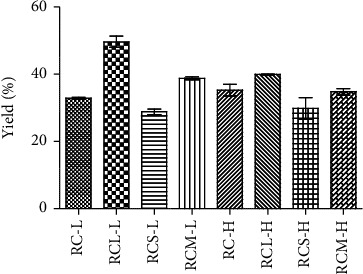
Yield percentage of each developed RUT-MP formulations. Two different RUT concentrations were employed; L annotated the lower concentration, while H annotated the higher concentration. Statistically, all yield data from individual RUT-MP formulations were significantly different one another (*p* < 0.05).

The yield percentages of all the developed formulations are presented in Figure 1. All the yield data were as follows: 32.9 ± 0.28; 49.6 ± 1.65; 28.8 ± 0.82; 38.8 ± 0.43; 35.2 ± 1.72; 39.9 ± 0.13; 29.8 ± 3.19; and 34.8 ± 0.96% for the respective formulations of RC-L; RCL-L; RCS-L; RCM-L; RC-H; RCL-H; RCS-H; RCM-H. The use of sugars as bulking agents improved the yield of spray drying (*p* < 0.05), except for SUC as its bulk density and their sticky nature might increased the stickiness of powder to the spray drying chamber [31]. The mass of dry powder collected after drying and the weight of all the solids in the feed are the two factors that determine product yield. Since a higher yield translates into more benefits, it is a crucial indicator for the industry. The stickiness issue with the formulation ingredients is the primary cause of the low product yield. However, there are techniques to enhance the product yield and, in turn, minimize, or lessen stickiness during spray-drying. Such techniques include mechanical scraping the drying apparatus, applying cold air from underneath, and employing low-temperature dehumidified air. Another strategy involves adding excipients with high glass transition temperatures to increase the feed's glass transition. In this case, the incorporation of sugars i.e., LAC, SUC, and MAL to the developed formulations exhibited benefits via the later strategy.

### 3.2. SEM

**Figure 2:**
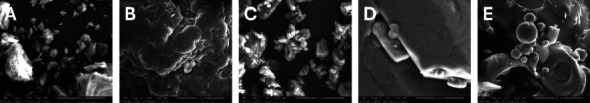
SEM images of pure materials: (A) RUT, (B) CAS, (C) LAC, (D) SUC, (E) MAL. Images were captured using magnification of 2500X.

SEM was used to examine powder morphology to look into how formulation and manufacturing aspects including air temperature at the inlet and outflow affected the particle morphology.  Figure 2 shows morphology of all the pure materials. The RUT, LAC, and SUC were crystal-like structure, whilst CAS and MAL were more to a noncrystalline form.

**Figure 3:**
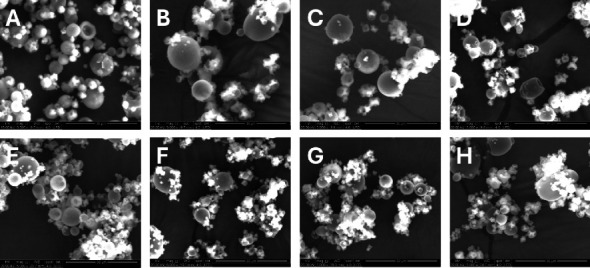
SEM images of the developed formulations at magnification of 5000x. The upper level is RUT-MPs with lower RUT concentrations: (A) RC-L, (B) RCL-L, (C) RCS-L, (D) RCM-L. The lower level is RUT-MPs with higher RUT concentrations: (E) RC-H, (F) RCL-H, (G) RCS-H, (H) RCM-H.

In comparison to the formulation without bulking agents, which displayed greater surface cave-ins, the powders with bulking agents had relatively smooth exterior shapes. As seen in Figure 3, the absence of bulking agents gives a more vacuole-like structure with fewer dimples on the surface of MP. Next, the addition of bulking agents reveals uniformity in the spray-dried powder's surface structure. The powder's rounded and smooth surface was noted for all formulations' particles that included bulking agents, regardless of lower or higher RUT concentrations. Bulking agents had a significant effect on the morphology of the MP and displayed amorphous-like physical properties. This significant alteration in surface morphology is consistent with the bulking agents superior glass properties, which have a high glass transition temperature.

### 3.3. XRD

**Figure 4:**
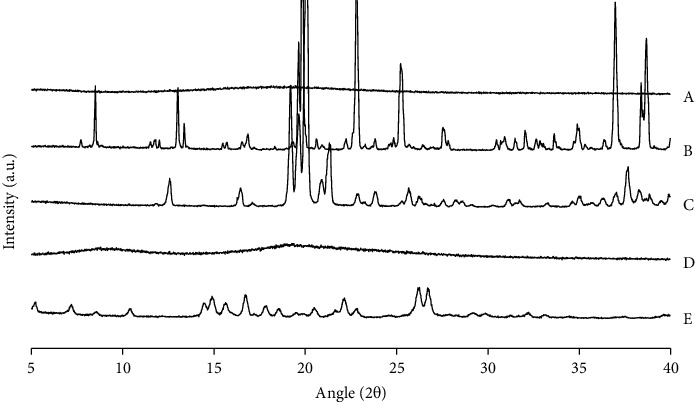
Diffractogram of pure materials: (A) maltodextrin, (B) sucrose, (C) lactose, (D) casein, and (E) rutin.

**Figure 5:**
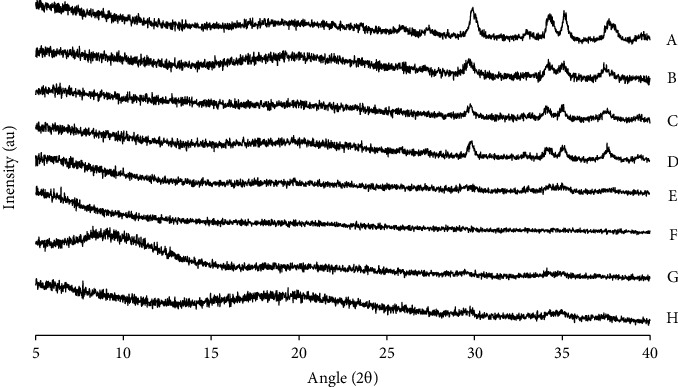
Diffractogram of the developed RUT-MPs formulations. Two different RUT concentrations were employed; L annotated the lower concentration, while H annotated the higher concentration. (A) RC-L, (B) RCL-L, (C) RCS-L, (D) RCM-L, (E) RC-H, (F) RCL-H, (G) RCS-H, and (H) RCM-H.

To further establish the physical state of RUT found in different samples, XRD patterns were investigated (Figures 4 and 5). The X-ray diffractograms of pure materials of RUT, LAC, and SUC exhibited several strong diffraction peaks between 5° and 40°, demonstrating those materials were highly crystalline structure (Figure 4). In contrast, the diffractograms of CAS and MAL showed no intensified peaks, implying the noncrystalline state of the two materials [26, 32].

A few diffraction peaks were also seen for the RUT-MP samples (Figure 5). Their intensity was comparatively lower than that of raw crystalline materials, which was explained by the lower content of the crystalline materials present in the RUT-MP samples. Such phenomenon was only observed in the formulations with lower RUT concentration without bulking agents (RC-L) and with bulking agents regardless of the type of bulking agents (RCL-L, RCS-L, and RCM-L). However, the thermograms of those samples were without any characteristic peaks of RUT, indicating that the encapsulation approach had caused RUT's crystalline structure transition to an amorphous state through the creation of MP, which was consistent with the DSC result. Interestingly, the formulations with higher concentration of RUT (RC-H, RCL-H, RCS-H, and RCM-H) exhibited no intensified peaks. This finding indicated that RUT crystallization was disrupted by encapsulating it in MP. This is advantageous for applying RUT to oral dosage form because the noncrystalline form of drug has a better bioavailability than the crystalline form.

### 3.4. DTA

**Figure 6:**
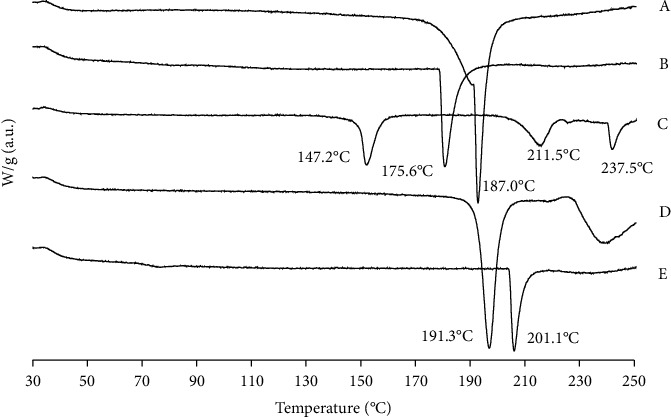
Thermogram of pure materials: (A) rutin, (B) casein, (C) lactose, (D) sucrose, and (E) maltodextrin.

DTA was used to assess the thermal characteristics of various samples to determine the physical state of RUT in MP (Figure 6). According to the thermogram, the pure RUT melting point was 187°C, suggesting the existence of crystalline RUT. The CAS showed an endothermic peak at 175.6°C [29]. All other pure ingredients were in crystalline forms indicated by relatively sharp peak melting points. LAC showed three endothermic peaks at 147.2°C, 211.5°C, and 237.5°C. The endothermic peak appeared at 147.2°C signifying the elimination of water of crystallization [33, 34]. This shows that the water of crystallization vaporizes out at the peak temperature. The next endothermic peaks around 211.5°C and 237.5°C, were the endotherm of the dehydrated LAC and subsequent α-lactose hydrate [35]. SUC was at 191.3°C and MAL was at 201.1°C [36, 37].

**Figure 7:**
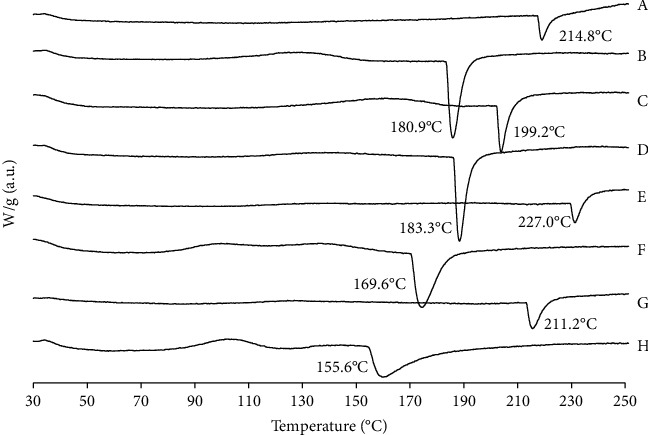
Thermogram of the developed RUT-MPs formulations. Two different RUT concentrations were employed; L annotated the lower concentration, while H annotated the higher concentration. (A) RC-L, (B) RCL-L, (C) RCS-L, (D) RCM-L, (E) RC-H, (F) RCL-H, (G) RCS-H, and (H) RCM-H.

The broad melting absorption peaks of the RUT-MPs varied among MP formulations (Figure 7). The absence of sugars in formulations regardless RUT concentrations of low (RC-L) or high (RC-H) showed peak at 214.8°C and 227.0°C, respectively. The two formulations exhibited no peaks associated with the melting of RUT crystals, indicating that the RUT no longer existed in crystalline form.

Nevertheless, all formulations that included sugars as bulking agents exhibited a single endothermic peak. The peak either slightly shifted to higher or lower temperature which was associated with either the sugar or the RUT crystal. For instance, formulations using LAC with lower RUT concentrations (RCL-L) showed peak at 180.9°C. This peak might correspond to a phase separated LAC itself as it corresponded to the proximity of the pure material. This was supported by XRD data. Formulation with higher RUT concentration (RCL-H) exhibited peak related with the LAC crystals at 169.6°C. Therefore, it was assumed that RUT was no longer present in crystalline form, and LAC was partially phase separated.

Different situation was found with the other formulations using SUC. The RCS-L and RCS-H formulations showed peak at 199.2°C and 211.2°C, respectively. Those peaks corresponded to the presence of SUC crystals without evidence of the peak associated with the melting of RUT. These results indicated that RUT behaved as an amorphous form in both formulations of RSC-L and RSC-H, which confirmed that RUT was successfully encapsulated in the MP.

The formulations using MAL showed a similar situation to those using LAC. The RCM-L and RCM-H formulations showed peak at 183.3°C and 155.6°C, respectively. The two formulations exhibited peaks associated with the melting of phase separated RUT crystals.

### 3.5. Solubility

**Figure 8:**
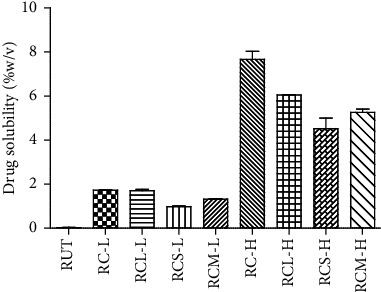
Solubility of pure RUT and its RUT-MPs formulations. The solubility data of RUT-MPs were significantly different with raw RUT (*p* < 0.05). Two different RUT concentrations were employed; L annotated the lower concentration, while H annotated the higher concentration. (RC-L) RUT:CAS, (RCL-L) RUT:CAS:LAC, (RCS-L) RUT:CAS:SUC, (RCM-L) RUT:CAS:MAL, (RC-H) RUT:CAS, (RCL-H) RUT:CAS:LAC, (RCS-H) RUT:CAS:SUC, (RCM-H) RUT:CAS:MAL.

By determining the pure RUT saturation concentration and from different RUT-MP formulations, the capacity of encapsulation to facilitate the solubility of RUT was assessed. The solubility of pure RUT and RUT from the RUT-MPs formulations were shown in Figure 8. The results extremely showed the poor solubility of RUT in water which is the major problem restricting its applicability (Table 2). Very little RUT was soluble (0.04% ± 0.01% b/v) and the color of the RUT-saturated solution was almost like pure water.

As presented in Table 2, the results showed that within the examined range, the RUT saturation was increased in the RUT-MP formulations compared to the pure RUT. The group of samples with lower RUT-containing formulations (RC-L, RCL-L, RCS-L, RCM-L) were 1.73 ± 0.01; 1.71 ± 0.06; 0.99 ± 0.03; and 1.34% ± 0.02% b/v, respectively. The formulations with higher RUT concentration (RC-H, RCL-H, RCS-H, RCM-H) were much higher with 7.66 ± 0.37; 6.05 ± 0.02; 4.52 ± 0.48; and 5.26% ± 0.15% b/v, respectively. All solubility data of RUT from the MPs formulations were significantly higher than those the pure RUT (*p* < 0.05).

The RUT solubilities from RUT-MPs formulations were significantly higher with 25 to 191,5-fold increases as compared to raw RUT (*p* < 0.05). As the RUT was encapsulated within the CAS, this formed a protective shell around it and enhanced its solubility by modifying its environment, preventing crystallization, and increasing its surface area [38, 39]. CAS has a unique amphiphilic structure that enhances solubility by trapping the hydrophobic RUT molecules in their core, shielding them from the aqueous environment and promoting better dissolution [27, 40]. The amorphous structure of RUT, as indicated by the XRD and DTA results, contribute to its improved solubility by increasing its surface area and reducing intermolecular forces, which is critical for improving its bioavailability and therapeutic efficacy. Such promising results worth further investigations on the dissolution properties of RUT in a simulated gastric fluid (SGF) with the gastric pH as well as simulated intestinal fluid (SIF) with the relevant pH.

## 4. Conclusion

RUT was successfully encapsulated in CAS in the form of RUT-MP. The solubility of the encapsulated RUT in the more basic environment (pH of 7.0 ± 0.05) was significantly increased by 190-fold compared to the raw RUT, which was achieved by formulation containing higher RUT concentration (RC-H). The use of sugar as bulking agents improved the yield of spray drying, except for SUC since its hygroscopicity might increased the stickiness of powder to the spray drying chamber. The RUT transformed from crystalline to amorphous structure as confirmed by DTA and XRD results. Overall, the results of this study revealed the improved solubility and potency of RUT-MP for further formulations into oral dosage forms such as tablets or capsules.

## Figures and Tables

**Table 1:** Prepared RUT-MP formulations for the enteric microparticles.

	RC-L (g)	RCL-L (g)	RCS-L (g)	RCM-L (g)	RC-H (g)	RCL-H (g)	RCS-H (g)	RCM-H (g)
RUT	0.25	0.25	0.25	0.25	1.25	1.25	1.25	1.25
CAS	2.50	2.50	2.50	2.50	2.50	2.50	2.50	2.50
LAC	—	2.50	—	—	—	2.50	—	—
SUC	—	—	2.50	—	—	—	2.50	—
MAL	—	—	—	2.50	—	—	—	2.50

*Note:* Two different RUT concentrations were employed; L annotated the lower concentration, while H annotated the higher concentration.

**Table 2:** Solubility data of raw RUT and RUT-MPs formulations.

Samples	Solubility (%w/v)
RUT	0.04 ± 0.01
RC-L	1.73 ± 0.015
RCL-L	1.71 ± 0.06
RCS-L	0.99 ± 0.03
RCM-L	1.33 ± 0.015
RC-H	7.66 ± 0.37
RCL-H	6.05 ± 0.02
RCS-H	4.52 ± 0.48
RCM-H	5.26 ± 0.15

## Data Availability

Data used in this study are available from the corresponding author on request.
